# A unique case report revealing the elusive clinical phenomenon of meningitis retention syndrome

**DOI:** 10.1016/j.eucr.2024.102885

**Published:** 2024-11-15

**Authors:** Sajal Gupta, Kishan Raj K, Prashanth Adiga K

**Affiliations:** Department of Urology & Renal Transplantation, Father Muller Medical College, Mangalore, Karnataka, 575002, India

**Keywords:** Meningitis. Urinary tract infection(UTI), Meningitis retention syndrome(MRS), Acute disseminated encephalomyelitis (ADEM)

## Abstract

Meningitis retention syndrome (MRS) represents a rare condition in which there is meningitis accompanied by urinary retention in the absence of any other neurological symptoms. MRS is usually misdiagnosed as a urinary tract infection due to varied clinical symptoms. It is a self-limiting syndrome that often presents with prodromal symptoms of meningitis. We report a case of MRS in a 26-year-old Indian male who presented clinically with features of meningitis and developed urinary retention during hospitalisation. Fewer than 30 cases have been described and several of them remain undiagnosed due to lack of diagnostic criteria. Any patient with fever and headache which are prodromal symptoms of meningitis followed by retention should be evaluated for MRS.

## Highlights

• This is a case on meningitis retention syndrome which is a rare and elusive thing in itself because it presents promoromaly to meningitis with retention of urine and mostly goes unnoticed and undiagnosed due to overlapping symptoms with UTI. This case will increase the horizon of the reader regarding this diagnosis and will trigger clinicians when they will encounter such patients.

## 1 Introduction

Acute urinary retention refers to the sudden inability to voluntarily void and is one of the common emergencies in urological clinics. Common causes of acute urinary retention include benign prostatic hyperplasia (BPH), constipation, urinary infections, sacral spinal cord diseases (Guillain- Barrè syndrome), aseptic meningitis, and cerebral demyelinating diseases (acute disseminated encephalomyelitis). Rare cases of acute urinary retention as a result of adverse drug reactions or post-surgical consequences have also been reported.^1^^,^^2^ These causes are common when compared to above mentioned neurological causes. Meningitis-retention syndrome (MRS) refers to a distinct presentation of acute urinary retention accompanied by meningitis, without any other neurological symptoms.^3^ This condition has not been well acknowledged, and hence its prevalence remains undetermined. To date, only 29 cases of MRS have been reported worldwide, and most of them were from Japan.^2^ Here we report a case of MRS in a 26-year-old Indian male patient.

## 2 Case presentation

A 26-year-old gentleman reported to the Department of General Medicine with the chief complaint of intermittent fever and headache for 5 days, which was associated with generalised weakness and myalgia. Patient also had a history of seizure while coming to the hospital. On general examination, the patient was febrile. All other vitals were within normal range. On systemic examination, terminal neck stiffness was observed. The patient was admitted with suspicion of meningitis. All the blood investigations were within normal range. Malarial parasite fluorescent, and Dengue Rapid test (NS1, IgM, IgG) was also negative.In view of suspected meningitis CSF analysis was done which showed 162 cells/cumm, glucose- 44 mg/dl, protein- 93 mg/dl, Amylase- 30 IU/L, and LDH- 77 IU/L. This was suggestive of pyogenic meningitis. On day one after admission to the hospital, the patient exhibited an episode of seizure and altered sensorium, due to which, he was shifted to the intensive care unit (ICU), and was started on the anti-epileptic medication, antibiotics, steroids and supportive treatment. Since the patient complained of headaches, seizures and altered sensorium magnetic resonance imaging (MRI) of the brain with time-of-flight (TOF) was done which showed a T2 hyperintense signal with diffusion restriction in the splenium of the corpus callosum. MRI Angiography of the Brain showed that all the surrounding arteries were normal in course and calibre. Electroencephalogram (EEG) showed generalised slowing, suggestive of diffuse cerebral dysfunction.

Patient complained of inability to void on the day 3 of admission. Transurethral catheterization was done using Foley's catheter and 600mL of urine was drained. Elaborated genitourinary examination of the patient revealed normal meatus, prostate was flat, firm, non-tender, with no nodule on palpation. Both bulbocavernosus reflex and cremasteric reflex could not be elicited on focussed neurological examination. Ultrasonography (USG) of the abdomen and pelvis revealed no significant abnormality. Patient was suspected to have UTI related retention but no abnormality was detected in urinalysis and urine culture showed no growth. The patient was started on alfuzosin 10mg. Following improvement in the patient's health status, he was shifted to the ward after 5 days. Later on, gradually the antibiotics and steroids were tapered and the patient was shifted to oral medications. The patient was discharged with a catheter on oral antiepileptics, protein supplements, laxatives and alpha-blockers.

**Fig. 1:**
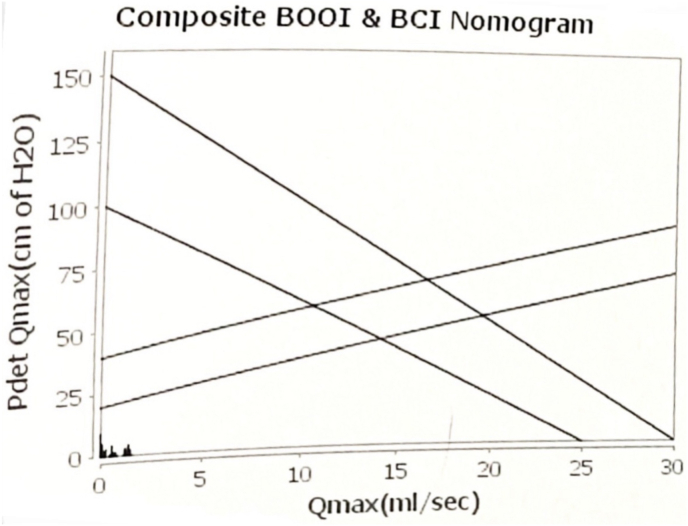
Composite BOOI AND BCI nomogram.

**Fig. 2:**
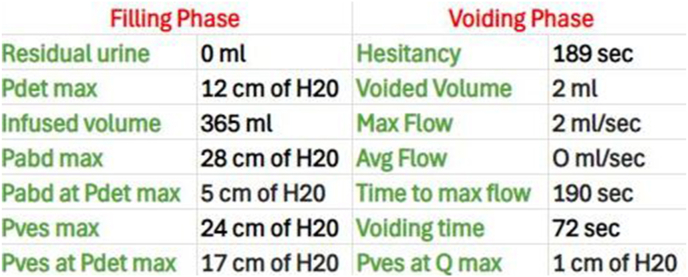
Urodynamic study findings.

At a 2-week follow-up visit, a urine culture was done, which came back negative. A trial void was given, but failed, hence the patient was again catheterized with Foley's catheter and was planned for urodynamic study(UDS). So after 2 weeks urodynamic study was done which revealed underactive detrusor activity with no voiding(Fig. 1, Fig. 2) hence was started on bethanechol 75 mg od and was advised clean intermittent catheterization.

**Fig. 3:**
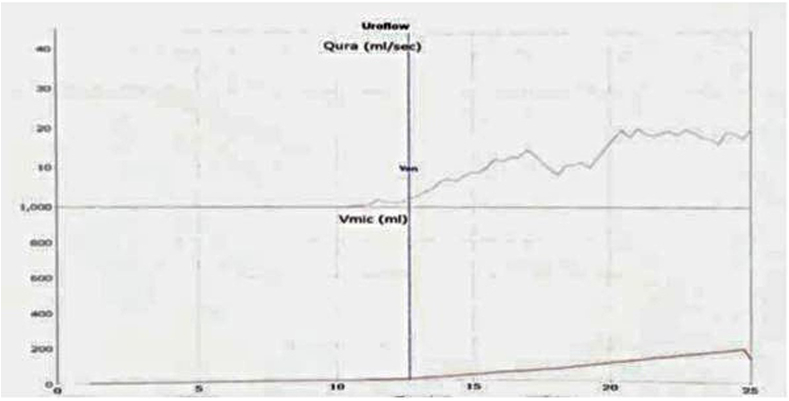
Uroflowmetry at 2-month follow-up.

However, the patient developed spontaneous voiding in between CIC in the follow-up period. So after 4 weeks, uroflowmetry was done showing voided volume 138ml Qmax flow 20 ml/sec (Fig. 3), which suggested complete recovery from the diseased condition. The patient has been under regular follow-up for 2 months without any complaints. ***This case report follows the principles of the Declaration of Helsinki. Informed consent has been obtained from participant and will be provided on demand.***

## 3 Discussion

We report a case of MRS in a 26-year-old male with fever and headache, who during hospitalisation for pyogenic meningitis, developed urinary retention. MRS was originally described by Sakakibara in 2005.^4^ The prevalence of MRS is under-reported, which may be because this condition is often misdiagnosed as urinary tract infection, due to its frequent association with abdominal pain. In MRS, there is no evident causative agent, and the specific symptoms and signs of meningitis are often absent. Thus, usually, the predominant presenting symptom is acute urinary retention alone.^4^ However, in our case, urinary retention developed after 1 week of the prodromal symptoms. Of 29 cases, in 14 cases, urinary retention developed within 1 week of prodromal symptoms.^2^

Based on the previously reported cases, the mean age of occurrence of MRS is 36 years, with a slight male predilection. In our case, the patient was a 26-year-old male. Commonly reported prodromal signs and symptoms include fever (86 %), and headaches (76 %), as was observed in our case, which was the presenting symptoms of the patient. Additionally, generalised weakness and myalgia were reported in our case. Meningeal symptoms were reported in 65 % of the previously reported cases of MRS, in our case we observed neck stiffness.^2^ Though the exact mechanism of MRS is not completely understood, it is speculated to occur as a result of spinal shock due to inflammation of upper motor neurons of the spinal cord, meningeal irritation, direct viral/bacteria entry, and the onset of acute disseminated encephalomyelitis after viral/bacteria infection.^5^^,^^6^ In our case, no viral etiology was found. It is presumed that bacterial or viral inflammation as well as post-infection inflammatory demyelination results in sacral myeloradiculopathy.^7^ MRI of the brain and spinal cord usually shows no abnormalities in MRS patients, which distinguishes it from acute disseminated encephalomyelitis (ADEM).^8^ This may be because the majority of cases are treated as meningitis, involving broad-spectrum antibiotics in combination with corticosteroids, and the use of corticosteroids might result in decreased inflammation, which in turn prevents the occurrence of cerebral and medullary lesions, which are characteristic of ADEM.^2^ However, in our case, an MRI brain with TOF showed a T2 hyperintense signal with diffusion restriction in the splenium of the corpus callosum.

In most of the cases of MRS, blood, urine, and CSF cultures are negative, which may suggest the autoimmune nature of MRS, thus supporting the hypothesis that MRS might be a mild form of ADEM, wherein medullary involvement is absent. In previously reported cases, CSF analysis has shown normal to mildly decreased glucose content, increased protein levels, and mild to severe lymphocytic pleocytosis. Sakakibara et al. have reported higher myelin basic protein levels in one patient, but no other cases have reported this parameter.^8^ However, in our case, CSF glucose and protein levels were within normal range.

A urodynamic study done in the majority of previously reported cases revealed an areflexic detrusor, resulting in an inability to contract the bladder for voiding.^2^ The urodynamic study performed in our case revealed an underactive detrusor with no voiding, due to which the patient was catheterized and bethanechol was given. Some hypotheses proposed for explaining detrusor hypofunction, and subsequent urinary retention in patients with MRS. Lesions of the central nervous system involving the brain or spinal cord may lead to detrusor hypofunction, as observed in cases of transverse myelitis or ADEM.^9^ However, features of myelitis and encephalitis are not present in MRS patients, and in such patients, neurologic etiology might be responsible for urinary retention. This is supported by the fact that urological abnormalities like infection of the urinary tract, have not been reported in any of the previous cases, including ours.^2^ Moreover, urinary retention was observed either concomitantly with meningitis or within a few days after meningitis, suggesting a strong chronological correlation. Though the exact site of the lesion remains questionable, meningeal irritation might lead to an initial acute spinal shock that compromises lower urinary tract innervation.

## 4 Conclusion

MRS is a rare disease condition, which is often misdiagnosed as a urinary tract infection. In this condition, meningitis is associated with urinary retention, and hence in patients presenting with fever and headache, MRS should be considered in the differential diagnosis and the patient should be kept under observation, since in most cases, urinary retention develops during the course of the disease, most often within one week. Medications that are helpful or not are still controversial. Although MRS is a self-limiting disease that resolves within a few weeks, in some cases, it may take 3–6 months.

## CRediT authorship contribution statement

**Sajal Gupta:** Conceptualization, Data curation, Investigation, Methodology, Visualization, Writing – original draft. **Kishan Raj K:** Conceptualization, Supervision, Writing – review & editing. **Prashanth Adiga K:** Conceptualization, Methodology, Supervision, Visualization.
